# Supplementary material for: Evaporation Driven Hydrovoltaic Generator Based on Nano-Alumina-Coated Polyethylene Terephthalate Film
Source: Polymers (Basel). 2023 Oct 13;15(20):4079. doi: 10.3390/polym15204079 (PMC10610091; doi:10.3390/polym15204079)
Supplement: Supplementary file 1 [file polymers-15-04079-s001.zip › Supplementary Materials.pdf]

Supplementary Materials

Evaporation Driven Hydrovoltaic Generator Based on Nano-alumina Coated Polyethylene Terephthalate Film

Shipu Jiao [1], Yihao Zhang [1], Yang Li [1], Bushra Maryam [1], Shuo Xu [1], Wanxin Liu [1], Miao Liu [1], Jiaxuan Li [1], Xu Zhang [1] and Xianhua Liu [1]*

[a]School of Environmental Science and Engineering, Tianjin University, Tianjin, 300354, PR China.

*Corresponding author. Tel.: +86-22-85356239; E-mail: lxh@tju.edu.cn

Table of contents

Figure S1. Preparation of the hydrovoltaic generator.

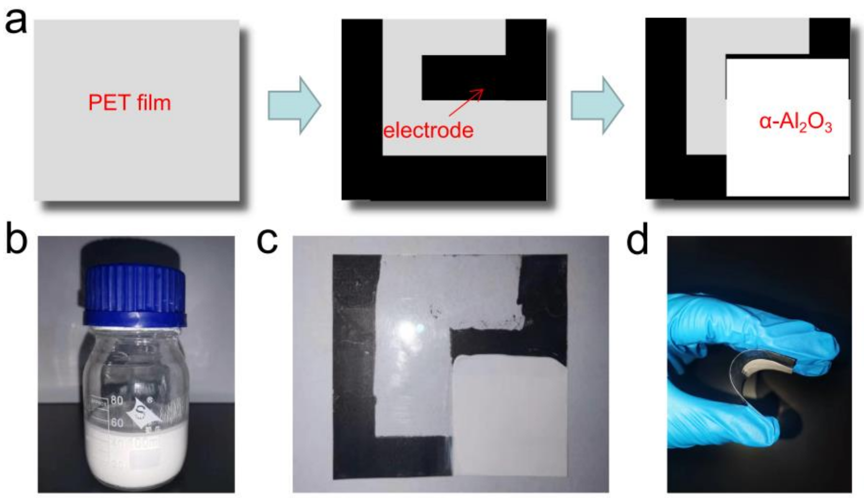

Figure S1. Preparation of the hydrovoltaic generator. (a) Schematic diagram of the preparation process of a hydrovoltaic generator; (b) Dispersion liquid made by dispersing alumina nanoparticles in ethanol; (c) Photo of an Al_2O_3-based hydrovoltaic generator; (d) A photo showing the excellent bending performance of the hydrovoltaic generator.

.
